# Risk Factors for Refractory Anaphylaxis in the Emergency Department

**DOI:** 10.1155/2024/9640278

**Published:** 2024-11-11

**Authors:** Ramiz Yazıcı, Hüseyin Mutlu, Ekrem Taha Sert, Kamil Kokulu, Ömer Faruk Turan

**Affiliations:** ^1^Department of Emergency Medicine, Kanuni Sultan Süleyman Training and Research Hospital, Istanbul, Türkiye; ^2^Department of Emergency Medicine, Aksaray University Medical School, Aksaray, Türkiye; ^3^Department of Emergency Medicine, Ankara Etlik City Hospital, Ankara, Türkiye

## Abstract

**Background:** Anaphylaxis is a serious allergic reaction that has a rapid onset and can result in death. Identifying the factors that trigger anaphylaxis and increase its severity is important for preventing refractory anaphylaxis (RA). In this study, we aimed to determine the factors associated with an increased risk of developing RA. Preventive measures to reduce the frequency and intensity of anaphylactic events are essential to provide the best care for allergic patients. Aggravating factors can trigger or increase the severity of anaphylaxis and therefore need to be recognized and avoided.

**Methods:** We retrospectively analyzed the data of 1378 patients over the age of 18 who were diagnosed with anaphylaxis in our clinic between January 1, 2020, and December 31, 2024. We divided the patients into two groups: anaphylaxis and RA. We evaluated the patients' clinical characteristics in the ED, demographic information, and elicitors that caused anaphylaxis.

**Results:** Of the 1384 anaphylaxis patients included in the study, 46 (3.3%) were diagnosed as RA. We determined that having a history of anaphylaxis is the most important determinant of the increased risk of RA. Having a history of anaphylaxis (OR: 2.87, 95% CI: 1.71–5.72), beta-blockers/ACEI use (OR: 2.47, 95% CI: 1.71–5.42), IV contrast agent (OR: 2.33, 95% CI: 1.64–5.39), and low blood pressure or related symptoms (OR: 2.34, 95% CI: 1.67–5.43) were more frequently associated with severe reactions.

**Conclusion:** We found that having low blood pressure or related symptoms, a known history of anaphylaxis, beta-blockers/ACEI, and IV contrast agent are risk factors for RA. To prevent mortality and morbidity in patients with this risk factor, early interventions such as rapidly repeating epinephrine doses and rapid fluid resuscitation should not be avoided.

## 1. Introduction

Anaphylaxis is an acute and fatal allergic reaction that occurs with non-homogeneous clinical scenarios depending on various triggering and enhancing factors [1]. The incidence of anaphylaxis is increasing globally every year, and the main causes are drugs, foods, and insect stings [2–4]. Signs and symptoms of anaphylaxis vary widely. Sudden skin or mucosal involvement (such as pruritus, urticaria, tongue, and uvula edema) is present in 90% of cases. Cardiovascular symptoms (cardiovascular collapse, hypotension, hypotonia, syncope, and urinary incontinence) affect more than 50% of patients. In addition, gastrointestinal involvement, including crampy abdominal pain, nausea, and vomiting, is observed in approximately 45% of the patients [3–7]. The estimated lifetime prevalence of anaphylaxis is 0.05%–2% and the mortality rate is 1%. Death mostly occurs as a result of upper or lower airway obstruction or cardiovascular collapse [6, 8]. Epinephrine, the only life-saving first-line drug in anaphylaxis, stabilizes mast cells and reverses almost all symptoms of anaphylaxis [1–5]. However, a single dose of epinephrine may be insufficient to stabilize patients in RA cases. RA can be defined as anaphylaxis that meets the criteria of the National Institute of Allergy and Infectious Diseases (NIAID) and the Food Allergy and Anaphylaxis Network (FAAN) [3] and does not improve in clinical symptoms after at least two doses of minimum intramuscular 300 *μ*g epinephrine treatment [5–10]. Treatment strategies for RA are different and more complex than normal anaphylaxis, and its frequency tends to increase in European countries [9, 10]. Additionally, the risk factors for RA are changing and are still unclear. It is important to recognize RA, whose treatment is different from normal anaphylaxis, and to determine its risk factors. In our study, we aimed to determine the frequency and risk factors of RA.

## 2. Materials and Methods

### 2.1. Study Design and Participants

We conducted this retrospective cohort study between January 1, 2020, and January 1, 2024, at the only tertiary care emergency department (ED) in the region (where an average of 30,000 patients were admitted per month). We received local ethics approval before the study (ethics committee number: 12/227085176). Patients over the age of 18 who applied to ED and were administered epinephrine at home, in the emergency ambulance service, or in the ED due to anaphylaxis were included in the study. Patients who were younger than 18 years of age, pregnant, diagnosed with hereditary angioedema, using antihistamines or steroids due to allergies, left the ED without completing the necessary treatment and follow-up, or those with missing records were excluded from the study.

### 2.2. Study Protocol and Data Collection

We obtained the patients' clinical and demographic characteristics, symptom onset time, follow-up notes, medications used, discharge notes, and all procedures performed in the ED from the hospital's medical electronic records. We divided those that cause anaphylaxis into four groups: foods, drugs, pesticides, and others. Patients diagnosed with anaphylaxis and whose clinical symptoms did not improve after at least two doses of intramuscular 0.5 mg epinephrine treatment were evaluated as RA. We divided the patients into two groups: anaphylaxis and RA.

### 2.3. Statistical Analysis

We presented descriptive statistics as frequency (*n*), mean ± standard deviation, median (25th–75th percentile), and percentage (%). We evaluated the consistency of the variables to normal distribution by the Kolmogorov–Smirnov test. We used Student's *T*-test for those with normal distribution and the Mann–Whitney *U* test for those without normal distribution. We compared categorical variables between the two groups using the chi-square test. We evaluated the relationship between clinical variables, time to onset of symptoms, conditions causing anaphylaxis, and RA using univariate logistic regression analysis. To identify predictors independent of parameters, we determined odds ratio (OR) and 95% confidence interval (CI) values and performed multivariate logistic regression analysis. We evaluated all statistical data using SPSS for Windows 22.0 software (Version 22.0; SPSS, Inc., Chicago, IL, USA). We performed the power analysis of the study using G∗Power 3.1 software. Since the study was retrospective, we accepted the type 1 error value as 0.01 and the effect size as 0.95 in the posthoc power analysis and calculated that the sample size of 46 people corresponded to a power of 1.0.

## 3. Results

**Figure 1:**
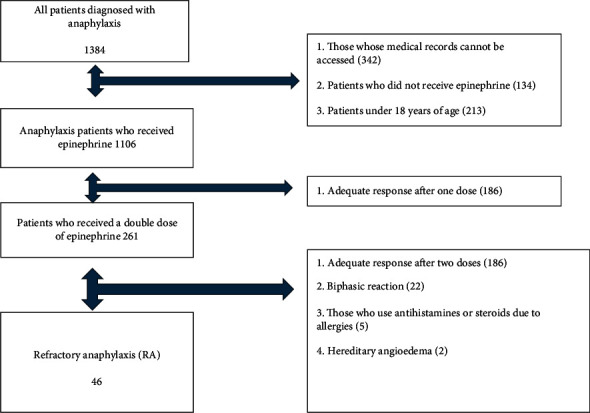
Study flowchart.

We included 1384 anaphylaxis patients who met NIAID/FAAN anaphylaxis criteria [4] in the study. We diagnosed 46 (3.3%) of the patients as RA. The flow diagram of the study is shown in Figure 1. The mean age of the anaphylaxis group was 39.2 ± 15.1 years. The mean age of the RA group was 44.3 ± 11.4 years and was older than the anaphylaxis group (*p* < 0.001). While 64.5% (863) of the patients in the anaphylaxis group were women, 67.3% of the patients in the RA group were women. A summary of the demographic data of the study population is shown in Table 1.

Variables that were found to be significant between anaphylaxis and RA as a result of univariate analyses, including clinical conditions, activating factors, and risk-increasing conditions for anaphylaxis, were included in the multivariate logistic regression model.

Among patients presenting with anaphylaxis, the probability of having RA was statistically higher in patients with a history of anaphylaxis (*p* < 0.001). Variables found to be significant between anaphylaxis and RA as a result of univariate analyzes were included in the multivariate logistic regression model. In multivariate logistic regression analysis, it was determined that having a history of anaphylaxis and using IV contrast/ACEIs were independent predictors of RA (Table 2).

## 4. Discussion

In anaphylaxis, rapid diagnosis and intramuscular epinephrine administration is required to prevent complications, including respiratory failure, cardiovascular collapse, and death, or to reduce the severity of the reactions [2–4]. Although anaphylactic reactions resolve immediately after a single dose of epinephrine in the majority of patients, patients may experience persistent or recurring symptoms that require additional interventions, as in RA. RA constitutes 3%–5% of all anaphylaxis cases in Europe and has been reported to be 9 times more common in the hospital environment compared to community reactions [11, 12]. Up to the present, many elicitors, clinical conditions, and drugs have been evaluated to predict RA. However, there is no single factor or condition sufficient for this. Risk factors for RA vary regionally. Therefore, we evaluated the risk factors for RA in anaphylaxis patients in our region. Our study is the first to evaluate the predictors of RA in our region.

We found that approximately 3 out of every 100 anaphylaxis patients did not respond to epinephrine administered according to the guidelines. We found that patients with RA were older than those with anaphylaxis, and the risk of RA increased 1.6 times for each age. We also found that having a history of anaphylaxis, beta-blockers/ACE, IV contrast medications, and low blood pressure or related symptoms were predictors of RA.

In a study conducted by the European Academy of Allergy and Clinical Immunology, it was suggested that older age is a factor associated with more severe reactions [3, 13]. Likewise, Van der Linden and colleagues reported that older age was associated with severe anaphylaxis in patients stung by insects [14]. In higher age groups, severe anaphylaxis is caused by increased cardiovascular instability [13–15]. Cardiovascular instability may be due to a reduced ability to activate mechanisms to prevent anaphylaxis due to conditions such as medications such as beta-blockers, peripheral vascular disease, or coronary artery disease [14, 15]. It may also be related to the increased use of medications (such as IV contrast) that are associated with the severity of anaphylaxis in the elderly [15]. Similar to the results of these studies, in our study, we found that the effect of age on RA increased by 1.63% (OR: 1.63, 95% CI: 1.23–4.97) for each year.

There is a strong relationship between RA and the increase in the levels of mast cell mediators, and it has been reported that the activation potential of mast cells decreases after antihypertensive drug treatment [1, 15, 16]. Nassiri et al. found that beta-blockers/ACEI is a risk factor for RA in both humans and rats and even their combined use further increases anaphylaxis [17]. We found that the likelihood of RA was higher in anaphylaxis patients using beta-blockers/ACEI and confirmed this in univariate or multivariate analysis (OR: 2.47, 95% CI: 1.92–5.41).

In a study conducted on 747 patients, Ewan et al. reported that having low blood pressure or related symptoms was an indicator of RA and that patients who had low blood pressure or related symptoms needed more treatment and follow-up than those who did not have [2–4, 18, 19]. In a study conducted in France, it was reported that patients referred to intensive care as a result of anaphylaxis had low blood pressure or related symptoms [20]. We similarly found that having low blood pressure or related symptoms was a risk factor for RA in anaphylaxis patients presenting to ED. The second most common cause of death due to drug-induced anaphylaxis in Australia is IV contrast agent [21, 22]. It also features prominently in recent studies from South Korea and Canada. Similarly, in the USA, it has been reported that IV contrast agent causes more fatal drug anaphylaxis than penicillin and cephalosporins combined [22–24]. The number of deaths in our study was low, but we found that IV contrast agent was a predictor for RA that could cause death (OR: 2.33, 95% CI: 1.64–5.39).

The most important strength of our study was the high number of patients. However, our study had some limitations. The first was that the study was conducted in a single center and the other was that the data were limited because it was a retrospective study. Our biggest limitation was that the patients in the study did not have tryptase levels. We evaluated only anaphylaxis patients admitted to ED. For this reason, patients who had anaphylaxis in the ward, intensive care unit, or at home were not included in our study. This may have led to an underestimation of mortality due to RA and anaphylaxis.

## 5. Conclusion

In conclusion, our study is one of the largest available studies evaluating the risk factor for RA in anaphylaxis patients admitted to ED. In our study, we found that having low blood pressure or related symptoms, a known history of anaphylaxis, and the use of beta-blockers/ACEI and IV contrast agent are risk factors for RA. To prevent mortality and morbidity in patients with this risk factor, early interventions such as rapidly repeating epinephrine doses and rapid fluid resuscitation should not be avoided. More studies are needed to identify patients at risk for RA and prevent fatal anaphylaxis.

## Figures and Tables

**Table 1:** Demographic information, clinical characteristics, and elicitors of patients presented to the ED with anaphylaxis.

Variables	Total anaphylaxis (*n* = 1384)	Anaphylaxis (*n* = 1338)	Refractory anaphylaxis (*n* = 46)	*p* value
Age (years)	40.1 ± 19.6	39.9 ± 7.6	45.2 ± 18.9	**< 0.001**
Sex (male), %	769 (%55.6)	743 (%55.5)	26 (%56.5)	0.546
Respiratory rate, breaths/min	20.0 ± 2	20.0 ± 2	22.0 ± 3	**< 0.001**
Oxygen saturation, %	97 (2)	97 (2)	96 (2)	0.136
Systolic blood pressure, mm Hg	112 (24)	112 (24)	107 (29)	**< 0.001**
Elicitor				
Foods, %	406 (30)	399 (30)	9 (19.5)	0.056
Drugs, %	294 (21)	272 (20)	22 (48)	**< 0.001**
Antibiotics, %	105 (7.6)	96 (7.1)	9 (19.5)	**< 0.001**
IV contrast agent, %	18 (1.3)	14 (1)	4 (8.6)	**< 0.001**
Muscle relaxants, %	54 (3.9)	52 (3.9)	2 (4.3)	**0.253**
NSAIDs, %	91 (6.5)	89 (6.7)	3 (6.4)	**0.017**
Insects, %	106 (7.7)	103 (7.4)	3 (6.7)	0.546
Bee venom, %	86 (6.2)	85 (6.3)	1 (2.1)	**< 0.001**
Yellow jacket venom, %	20 (1.4)	18 (1.3)	2 (4.3)	**< 0.001**
Others or unknown, %	378 (27.3)	366 (27.3)	12 (46)	0.051
Clinical criteria for anaphylaxis				
Skin–mucosal involvement∗	1352 (98)	1307 (98)	45 (98)	0.546
Respiratory compromise•	1017 (73)	966 (76)	41 (89)	**< 0.001**
Reduced BP or associated symptoms+	169 (12)	140 (11)	29 (63)	**< 0.001**
Gastrointestinal symptoms‡	309 (22)	296 (22)	14 (30)	**< 0.001**
Comorbidities				
Asthma	267 (19)	257 (19)	10 (22)	**0.017**
Diabetes	24 (1.7)	23 (1.7)	1 (2.1)	0.546
Cardiologic disease	45 (3.5)	39 (2.9)	6 (13)	**< 0.001**
Infection	78 (5.6)	76 (5.6)	2 (4.4)	0.062
History of malignant disease	44 (3.1)	42 (3.1)	2 (4.4)	0.246
Mastocytosis	8 (0.5)	8 (0.5)		
Exercise prior to reaction	105 (7.6)	103 (7.7)	3 (6.6)	0.089
Concomitant medication				
ASA	57 (4.1)	49 (3.7)	8 (17)	**< 0.001**
Beta-blockers/ACEI	49 (3.5)	39 (2.9)	10 (21)	**< 0.001**
PPI	134 (9.6)	130 (9.7)	4 (8.7)	0.186
History of anaphylaxis, *n* (%)	287 (21)	263 (20)	24 (52)	**< 0.001**
Duration of stay in the ED (minutes)	372 ± 76	370 ± 76	512 ± 106	**< 0.001**
Hospitalization	104 (7.5)	58 (4.3)	46 (100)	**< 0.001**
Patient died			1	

*Note:* Significance value *p* < 0.05 for values in bold.

^∗^Generalized hives, itch-flush, and swollen lips–tongue–uvula.

^•^Dyspnea, wheeze-bronchospasm, stridor, reduced PEF, and hypoxemia.

^+^Hypotonia (collapse), syncope, and incontinence.

^‡^Crampy abdominal pain and vomiting.

**Table 2:** Univariate and multivariate analysis of predictive factors for RA.

Variables	Univariate logistic regression		Multivariate logistic regression	
	OR (95% CI)	*p* value	OR (95% CI)	*p* value
Age (per 1 year)	1.95 (1.68–3.98)	< 0.001∗	1.63 (1.23–4.97)	0.042∗
Respiratory rate, breaths/min	1.59 (1.36–2.83)	0.015	1.17 (1.04–4.92)	0.113
Systolic blood pressure	2.04 (1.29–4.22)	< 0.001∗	1.54 (1.21–5.78)	0.064
Antibiotic	1.13 (1.04–2.64)	0.041	1.07 (0.89–3.56)	0.276
IV contrast agent	2.07 (1.94–4.58)	< 0.001∗	2.33 (1.64–5.39)	< 0.001∗
Bee venom	1.09 (1.02–2.04)	0.051	1.07 (0.87–3.06)	0.276
Yellow jacket venom	1.21 (1.05–1.41)	0.034	1.13 (1.05–2.90)	0.346
Respiratory compromise	1.27 (1.11–1.47)	0.031	1.39 (1.15–4.86)	0.091
Reduced BP or associated symptoms	2.08 (1.61–3.89)	< 0.001∗	2.34 (1.67–5.43)	< 0.001∗
Cardiologic disease	1.63 (1.22–3.17)	0.011	1.19 (1.07–1.68)	0.225
Gastrointestinal symptoms	1.52 (1.19–2.86)	0.013	1.28 (1.16–1.34)	0.074
ASA	1.88 (1.34–3.58)	< 0.001∗	1.93 (1.64–5.39)	0.01∗
Beta-blockers/ACEI	2.59 (1.96–5.63)	< 0.001∗	2.47 (1.92–5.41)	< 0.001∗
History of anaphylaxis	2.19 (1.86–4.98)	< 0.001∗	2.87 (1.71–5.62)	< 0.001∗

Abbreviations: ACEI, angiotensin-converting enzyme inhibitor; ASA, acetyl salicylic acid; BP, blood pressure; CI, confidence interval; OR, odds ratio; RA, refractory anaphylaxis.

^∗^ *p* value < 0.05.

## Data Availability

The data used to support the findings of this study are available on request from the corresponding author.
